# Adrenomedullin as a Growth and Cell Fate Regulatory Factor for Adult Neural Stem Cells

**DOI:** 10.1155/2012/804717

**Published:** 2012-09-24

**Authors:** Sonia Martínez-Herrero, Ignacio M. Larráyoz, Laura Ochoa-Callejero, Josune García-Sanmartín, Alfredo Martínez

**Affiliations:** Oncology Area, Center for Biomedical Research of La Rioja (CIBIR), 26006 Logroño, Spain

## Abstract

The use of stem cells as a strategy for tissue repair and regeneration is one of the biomedical research areas that has attracted more interest in the past few years. Despite the classic belief that the central nervous system (CNS) was immutable, now it is well known that cell turnover occurs in the mature CNS. Postnatal neurogenesis is subjected to tight regulation by many growth factors, cell signals, and transcription factors. An emerging molecule involved in this process is adrenomedullin (AM). AM, a 52-amino acid peptide which exerts a plethora of physiological functions, acts as a growth and cell fate regulatory factor for adult neural stem and progenitor cells. AM regulates the proliferation rate and the differentiation into neurons, astrocytes, and oligodendrocytes of stem/progenitor cells, probably through the PI3K/Akt pathway. The active peptides derived from the AM gene are able to regulate the cytoskeleton dynamics, which is extremely important for mature neural cell morphogenesis. In addition, a defective cytoskeleton may impair cell cycle and migration, so AM may contribute to neural stem cell growth regulation by allowing cells to pass through mitosis. Regulation of AM levels may contribute to program stem cells for their use in medical therapies.

## 1. Introduction

A continuously increasing number of research articles reporting new experimental data on stem cells confirm the trend that began in 1999. Due to the prospects for the translation of stem cell biology advancements to treatment of many severe conditions such as Parkinson disease [1, 2], Alzheimer disease [3], Duchenne muscular dystrophy [4], amyotrophic lateral sclerosis [3], diabetes [5], stroke [6], myocardial regeneration [7], cartilage repair [8], or acute fail liver [9], stem cells are common in the popular press and great hopes are stirring in the public about their therapeutic potential.

The dogmatic view of an ever-immutable neural tissue in mammals is now been replaced by the notion that cell turn over does occur in the mature central nervous system thanks to the persistence of precursor cells that possess the functional characteristics of neural stem cells [10]. In modern society, where neurodegenerative diseases are becoming a major public health problem, neural stem cells have become one of the main attention points of the scientific community. Their capacity to self-renew and to produce all cell types of the mature central nervous system leads to hypothesize about their potential use in transplantation therapies for severe neurodegenerative diseases such as Parkinson or Alzheimer disease [4].

All the processes of self-renewal, proliferation, progressive maturation, and differentiation, which are needed for stem cell physiology, are orchestrated by a set of transcription factors, cell-to-cell interactions, niche-to-cell interactions, and many soluble diffusible signals [11]. One of these signals is adrenomedullin (AM), a 52-aminoacid peptide with structural homology to calcitonin gene-related peptide [12, 13]. AM displays a large variety of physiological functions including cell growth and differentiation regulation [14]. In addition, recent studies point to specific roles of this regulatory peptide in the behaviour of several stem cells, including neural stem and progenitor cells. This paper tries to sum up the current knowledge about this topic. 

## 2. Adrenomedullin

This regulatory peptide was isolated from human pheochromocytoma by Kitamura et al. in 1993 [15]. This peptide was able to stimulate cAMP production in human platelets and exerted potent and long-lasting hypotensive activity in rats. AM is synthesized by both tumor cells and normal adrenal medulla as well as by many other tissues. AM is a circulating hormone, although it functions also as a local paracrine and autocrine mediator with multiple biological activities such as vasodilatation, cell growth, regulation of hormone secretion, natriuresis, and antimicrobial effects [16]. 

### 2.1. Structure of Adrenomedullin

Human AM consists of 52 amino acids and it belongs to the amylin/calcitonin gene-related peptide (CGRP) family. Intermedin, also named adrenomedullin 2, has also been identified as a novel member of this family [12, 13]. AM contains a 6-amino acid ring formed by a disulfide bond between residues 16 and 21. The C-terminal tyrosine residue is amidated (-CONH_2_). Both structural features are essential for its biological activity [17]. 

The three-dimensional structure of AM comprises a central *α*-helical region, covering approximately one third of its total length, flanked by two disordered segments. The presence of the *α*-helix at the centre of AM seems to be a general feature of the calcitonin peptide family, which is important for the physiology of these peptides and the recognition of their specific receptors [18]. 

### 2.2. Adrenomedullin Gene Expression and Release

AM is encoded by a gene contained in human chromosome 11 and consisting of 4 exons and 3 introns, with TATA, CAAT, and GC boxes in the 5'-flanking region [16]. In mice, the AM gene is localized in chromosome 7. 

The mature AM peptide is derived from preproadrenomedullin, which contains 185 amino acids in humans. After cleaving of 21-residue N-terminal signaling peptide, preproadrenomedullin is converted to proadrenomedullin, which is a precursor of mature AM (amino acids 95-146 of preproadrenomedullin) as well as of another active peptide, proadrenomedullin N-terminal 20-peptide or PAMP (amino acids 22-41 of preproadrenomedullin) [17].

AM production is mostly regulated by oxidative stress and inflammation-related substances such as lipopolysaccharide and inflammatory cytokines like TNF-*α* and IL-1, which increase AM secretion rate. There are several binding sites for activator protein-2 (AP-2) and c-AMP-regulated enhancer element. It has also been discovered that there are nuclear factor-K*β* sites on the promoter of the AM gene [14]. Hypoxia is also a potent inducer of AM expression. This overexpression is mediated by transactivation of the AM promoter by hypoxia inducible factor 1 (HIF-1) transcription factor, as well as by posttranscriptional mRNA stabilization. Hypoxia response elements (HREs) have been found in the promoter of the human AM gene [19]. 

### 2.3. Metabolism of Adrenomedullin

AM is a circulating peptide and it can be found in plasma at a concentration of 2–10 pM in humans. AM is also present in other biological fluids such as urine, saliva, sweat, milk, amniotic fluid, and cerebrospinal liquid [16]. In plasma, AM is specifically bound to adrenomedullin binding protein-1 (AMBP-1), which was later identified as complement factor H [20]. AM bound to complement factor H cannot be detected in plasma, so it is thought that total plasma AM could be higher than reported in most studies. Circulating AM is rapidly degraded with a halflife of 16–20 minutes. Matrix metalloprotease 2 seems to be responsible for the initial degradation of AM, which is followed by an aminopeptidase [21, 22].

### 2.4. Adrenomedullin Receptor Distribution

Specific binding sites for AM are located in many cell types and tissues like the heart, lungs, spleen, liver, vas deferens, kidney glomerulus, skeletal muscle, hypothalamus, spinal cord, and so on [16]. The wide distribution of binding sites for AM has to be related with its great variety of biological functions. In addition, recent studies reveal that AM is able to bind to many areas of the brain, providing the anatomical basis for the involvement of AM in the physiology of the central nervous system (CNS) [23]. 

The same studies also provide evidence for the possible existence of heterogeneous populations of AM binding sites in the brain and peripheral tissues [23] with different affinities for AM. 

The AM receptor belongs to the 7-transmembrane domain G-protein-coupled receptor superfamily and is named calcitonin receptor-like receptor (CLR). However, CLR needs the presence of modulating proteins with a single transmembrane domain known as receptor activity modifying proteins (RAMP) [24]. Three RAMP isoforms have been identified in the human genome: RAMP1, RAMP2 and RAMP3. RAMPs bind to the CLR in the endoplasmic reticulum promoting the plasma membrane localization [25]. RAMP1 transports and present the CLR at the membrane surface as a mature glycoprotein, and this heterodimer functions as a CGRP receptor. The CLR molecules transported by RAMP2 and RAMP3 are core-glycosylated and function as AM receptors (AMR); CLR/RAMP2 is known as AMR_1,_ whereas CLR/RAMP3 is AMR_2_ [26].

Qi et al. [27] studied the sequence differences between RAMP1, RAMP2, and RAMP3 to identify individual residues that could be able to alter their pharmacology. They hypothesized that residues present in RAMP2 and RAMP3 but not in RAMP1 are responsible for making CLR/RAMP2 and CLR/RAMP3 AM receptors. It was reported that position 74 in RAMP2 and RAMP3 is critical to establish their affinity for AM while Phe93 in RAMP1 contributes in a very important way to *α*CGRP affinity for CGRP receptors [27]. 

The expression of RAMP isoforms in a particular cell may change between physiological and pathological conditions, determining the degree of response to AM and CGRP. In physiological conditions the more abundant isoform is RAMP2, suggesting that most CLR molecules bind to RAMP2 to form a functional receptor for AM. Despite this observation, dynamic changes in the expression pattern of RAMP genes may take place during disease and in some physiological conditions like pregnancy. The most robust changes in RAMP expression levels coincide with those situations in which plasma AM is most elevated, as in pregnancy or diseases like sepsis or heart failure. In those situations in which AM levels are higher, there is an elevation in RAMP3 expression apparently in order to decrease AM responsiveness [28]. 

### 2.5. Signal Transduction Mechanisms

The signal transduction pathways activated by AM vary between species, organs, tissues, and cells. However, there are three main signaling pathways whereby AM exerts its actions: cAMP, Akt, and mitogen-activated protein kinase (MAPK) extracellular signal-regulated protein kinase (ERK).

The main signal transduction pathway activated by AM seems to be the adenylyl cyclase/cAMP system. In many cell types, AM and CGRP receptors are coupled to G_s_ proteins that activate adenylate cyclase and increase intracellular levels of cAMP [17]. In bovine aortic endothelial cells and vascular smooth muscle cells (VSMC), the accumulation of cAMP causes the activation of protein kinase A (PKA) which in turn increases calcium (Ca^2+^) efflux leading to relaxation of the vascular cells [29]. 

Moreover, AM can induce Ca^2+^ mobilization independently from cAMP levels. This fact suggests that other signalling mechanisms may be responsible for AM-induced positive ionotropic action. This theory was confirmed by Szokodi et al. [30]. AM activated phospholipase C through its specific receptor and accelerated Inositol-1,4,5-P_3_ formation to stimulate Ca^2+^ release from the endoplasmic reticulum intracellular stores [29, 30]. In addition, the activation of phospholipase C is also involved in ion channel opening. However, other studies have shown that AM administration does not have any effects in intracellular Ca^2+^ concentration and even decreases Ca^2+^ content in cultured HUVECs [31] or in porcine coronary arteries [32]. These results suggest that the regulation of Ca^2+^ mobilization by AM may depend on the cell type and context. 

It has been shown that intracellular Ca^2+^ increases, in response to AM, caused activation of nitric oxide (NO) synthase and NO release leading to relaxation of cardiac myocytes [33]. AM activation of NO pathway has a very important role in the regulation of the cardiovascular system by regulating blood flow [34]. The NO pathway and the reactive oxidative species produce an elevation of collateral flow in ischemic tissues having a cytoprotective action against ischaemia/reperfusion injury and against myocardial ischaemia-induced arrythmias in rats [35]. Furthermore, Sata et al. demonstrated that AM inhibited endothelial cell apoptosis through a NO-dependent pathway [36]. Their results suggest that the antiapoptotic effect of AM mediated by NO is independent of cGMP, whereas the cGMP/cGMP-dependent kinase pathway mediates many biological functions of NO-like vasodilatation [36]. Some authors postulate that NO prevents apoptosis by S-nitrosylating caspases [37–39]. 

AM has been shown to activate the PI3K/Akt pathway in vascular endothelial cells where it regulates many steps like vasodilation, cell survival, proliferation, migration, and vascular cord-like structure formation [40]. The specific role of AM in the multistep process of angiogenesis is regulated via a mechanism that requires the activation of the CLR/RAMP2 and CLR/RAMP3 receptors [41]. 

Other findings suggest that AM also acts directly on myocardium by the presence of CLR in myocytes and induces cardioprotective and antiapoptotic effects through the activation of the PI3K/Akt pathway after ischemia/reperfusion and enhances neovascularization in ischemic tissues [42]. 

The role of AM in growth and mitogenesis has led to investigate the regulation of MAPK by AM. AM signalling directly promotes endothelial cell growth and survival through activation of MAPK/ERK downstream signalling pathways [43]. Interestingly, in glomerular mesangial cells AM causes an opposite effect by increasing apoptosis during serum deprivation [44]. AM activates apoptosis in mesangial cells but protects against it in endothelial cells and tumors; these results suggest a cell type-dependent effect of AM on apoptosis [14]. Activation of MAPK and other kinases such as cAMP-PKA, JNK, and protein phosphatase 2A (PP2A) has been proposed to mediate the proapoptotic effect of AM in mesangial cells. On the other hand, AM protects malignant cells from hypoxia-induced cell death by upregulation of Bcl-2 in an autocrine/paracrine manner [45]. 

AM appears to either stimulate or inhibit cell proliferation depending on the particular cell type. Cell proliferative response induced by AM is thus mediated via activation of protein tyrosine kinase-MAPK. However, in cells lacking MAPK activation signalling pathways, stimulation of AM receptors results in activation of cAMP, leading to growth inhibition [46]. 

On vascular smooth muscle cells (VSMCs) there are conflicting results. Initially it was suggested that AM had an antiproliferative effect [47]. But more recent studies suggest that AM exerts a potent mitogenic effect in serum-deprived VSMCs [48, 49]. Under serum deprivation, AM promotes DNA synthesis and cell proliferation in VSMCs. These responses are mediated by p42/p44 MAPK activation. AM stimulates proline-rich tyrosine kinase 2 (PYK2) which, in turn, activates c-Src and induces recruitment of adaptor proteins (Shc/Grb2), thereby leading to activation of the Ras-dependent MAPK cascade in VSMCs.

All signal mechanisms in which AM is involved are the basis of its extensive repertoire of biological functions as vasodilation, cellular proliferation, apoptosis modulation, or inflammatory regulation. 

The main role played by AM in mammalian development has become apparent following the generation of different knockout (KO) models. In AM gene KO mice, in which the expression of AM and PAMP are suppressed, the null phenotype is embryonically lethal due to the absence of placental vascularisation, malformation of the basement membrane in the aorta and cervical arteries, detachment of the endothelial cells from the basement structure, and the presence of edema [50]. In addition, other groups have generated a KO mouse model in which only AM expression is affected [51]. AM^−/−^ mice are also embryonic lethal and both genotypes AM/PAMP^−/−^ and AM^−/−^ caused embryonic lethality at the same embryonic age, between embryonic day 14 (E14.5) and embryonic day 15 (E15.5).

To demonstrate the *in vivo* importance of the CLR, a gene-targeted KO model of its gene (Calcrl) was generated. Mice heterozygous for the targeted Calcrl allele appear normal, survive to adulthood, and reproduce normally. However, Calcrl^−/−^ pups are not viable, the embryos die between E13.5 and E14.5 of gestation and they exhibit a very similar phenotype to AM^−/−^ and AM/PAMP^−/−^ mice [52]. This result demonstrates that Calcrl is essential for embryo survival.

In models of mice lacking RAMP2 the results are similar to the ones shown above. RAMP2^−/−^ embryos die in utero at midgestation due to severely deformation, vascular fragility, severe edema, and hemorrhage. In contrast, RAMP2^+/−^ mice are viable, they reach adulthood but they exhibit a great variety of phenotypes due to vascular hyperpermeability and impaired neovascularization [53]. These data show that RAMP2 is a key determinant of the effects of AM on the vasculature and is essential for angiogenesis and vasculature integrity. 

Surprisingly, a complete absence of RAMP3 has no effect on survival. RAMP3-null mice appear normal until old age (9-10 months), at which point they have less weight than their wild-type littermates [54]. These results provide support to the hypothesis that RAMP2 and RAMP3 have distinct physiological functions in embryogenesis, adulthood, and old age.

To continue with the study of the lack of AM in adult tissues and organisms, tissue-specific conditional KO models have been generated using Cre/loxP technology [55]. For example, loss of AM receptor resulted in abnormal jugular lymphatic vessels in association with reduction in lymphatic endothelial cell proliferation [43]. Another report, by Fernández et al., showed that lack of AM in the mouse nervous system results in behavioural changes, anxiety, and lower survival under stress conditions [56]. In addition, AM suppression causes significant infarct size increase following cerebral artery occlusion [57]. 

### 2.6. Physiological Activity of Adrenomedullin

In the adult organism, AM has been located in many cell types and in most tissues throughout the body [58]. This distribution suggests that AM has diverse physiological activities and that it needs a tight regulatory system. The locations of AM expression include the nervous system and related structures, cardiovascular system, endocrine organs, digestive tube, excretory system, respiratory system, reproductive tract, and integument, among others.

AM has a variety of biological actions which are of potential importance for cardiovascular homeostasis, growth and development of cardiovascular tissues, and regulation of body fluid. Systemic AM administration has demonstrated that this peptide reduces arterial pressure, decreases peripheral vascular resistance, and increases heart rate and cardiac output [59]. *In vitro, *AM dilates blood vessels of different vascular beds from different animal species. Regarding the mechanism of the vasodilatory effect of AM, most data indicate that AM may induce endothelium-independent relaxation by acting on CGRP_1_ receptors and elevating cAMP level in vascular smooth muscle cells [17]. In addition, AM binds to specific receptors in endothelial cells and elicits endothelium-dependent vasorelaxation mediated by nitric oxide [60], endothelium-derived hyperpolarizing factor [61], and/or vasodilatory prostanoids [62]. On the other hand, accumulating evidence supports a compensatory role for AM in heart failure. It has been established that plasma AM levels increase in patients with heart failure in proportion to the severity of the disease. Furthermore, recent studies suggest that plasma AM level is an independent prognostic indicator of heart failure. This peptide may regulate myocardial hypertrophy and remodelling in arterial hypertension or heart failure in a paracrine/autocrine way [63]. 

AM exerts a tight control on renal function and body fluid volume. First, AM exerts direct control over the kidney, affecting diuresis and natriuresis. Furthermore, AM may regulate the hypothalamic-pituitary-adrenal axis at all levels. It has been demonstrated that intracerebral infusions of AM inhibit salt intake and thirst. In the pituitary, AM is able to inhibit the secretion of vasopressin and ACTH, whereas in the adrenal gland it regulates the secretion of aldosterone [64]. 

AM regulates hormone secretion in many tissues and organs. Levels of this peptide have effects in the hypothalamic-pituitary-adrenal axis as shown above. In addition, AM is synthesized in pancreatic polypeptide-producing F cells of the pancreatic islets and AM receptors are expressed in insulin-producing *β*-cells. Incubation with a monoclonal antibody against AM raised five times the basal insulin secretion by islets [65]. This indicates that endogenous AM tonically inhibits insulin secretion. 

In the reproductive tract, AM is synthesized by the granulosa cells of the ovarian follicles. Plasma AM level increases during the follicular phase and decreases during the luteal phase of the menstrual cycle [66]. Both AM and its receptors are expressed in the uterus and their expression is higher during pregnancy [67]. AM can also be detected in the placenta and in many fetal tissues suggesting that it may be involved in growth and embryogenesis [68]. 

In the digestive system AM immunoreactivity is widely distributed in the mucosal and glandular epithelia of the stomach, esophagus, intestine, gallbladder, bile duct, and acini of the pancreas and salivary glands [69]. AM is a potent inhibitor of basal gastrin-stimulated HCl secretion [70].

Finally, AM has been found in all epithelial surfaces which separate the external and internal environment and in all body secretions. This wide distribution suggests the possibility that AM has an immunity-related function. It has been proven that both AM and PAMP display potent antimicrobial action against Gram-positive and Gram-negative bacteria [71]. In septic shock patients, a marked elevation of AM blood levels have been reported, probably as a defensive action [72]. However, excessive AM release during septic shock may provoke adverse effects such as hypotension which may threaten the patient's life.

## 3. Adrenomedullin in the Central Nervous System

AM and its receptors are abundantly expressed in the CNS and its cellular components. By a combination of techniques it has been proven that AM appears from day 10 of embryonic development in maturing cells of the ventral horn of the spinal cord and by day 14 mRNA for AM and its receptors could be observed in specific neuron groups [73]. This was followed by the demonstration of the presence of human AM mRNA in the following regions: frontal, temporal and occipital cortex, pons, thalamus, hypothalamus, cerebellum, and in the pituitary gland [74]. With respect to the rat cerebral cortex, AM immunoreactivity was shown to be widely distributed in all areas under basal conditions. In particular, high levels of immunoreactivity were found in the thalamus, hypothalamus, adenohypophysis, and neurohypophysis [75]. 

Given the wide distribution of AM immunoreactivity in the adult CNS, it has to be admitted that the function of AM at most of these sites is still largely unknown. It is apparent that AM can act as a neurotransmitter, neuromodulator or neurohormone; but in sight of its ample distribution, it could have other functions apart from those. The prominent localization of AM in dendritic structures and the cytoskeleton also argues against an exclusive role as a neurotransmitter [75].

The AM system may be especially important in cerebral circulation. The concentration of AM is about 50% higher in cerebral blood vessels than in other regional circulations due to an astrocyte-induced elevation of AM production by cerebral endothelial cells [76]. However, the biological actions of AM in cerebral blood vessels have only been partially defined. 

AM, as an endothelium-derived autocrine/paracrine hormone, plays an important role in the regulation of specific blood-brain barrier properties [77]. AM may be one of the physiological links between astrocyte-derived factors, cAMP and the induction and maintenance of the blood-brain barrier. Moreover, the role of AM in the differentiation and proliferation of peripheral endothelial cells and in angiogenesis suggests a more complex function for AM in the cerebral circulation and in the development of blood-brain barrier [78]. 

The hypotensive effect of AM in the periphery is not paralleled in the brain. Both, AM and PAMP, given by intracerebroventricular (icv) infusions, exerts significant hypertensive actions in conscious animals by a central action which stimulates *α*-adrenergic, sympathetic function. In addition to increasing blood pressure after icv administration, both peptides stimulated significant increases in heart rate in these conscious, unrestrained animals [79]. Direct effects of AM centrally administered on sympathetic nerve activity in conscious rats were observed and these data suggest that icv AM induced an increase in preganglionic sympathetic discharge [80]. The temporal aspects of CNS-induced hypertension by AM are similar to those effects seen after the administration of angiotensin II [79, 80]. This suggests a possible common mechanism for the hypertensive action of both peptides in the brain. 

Regarding cerebral physiopathology, using the rat focal stroke model of middle cerebral artery occlusion (MCAO), Wang et al. [81] showed increases in AM mRNA expression in the ischemic cortex. Immunohistochemical studies localized AM to ischemic neuronal processes. However, this study reports that AM exacerbates focal brain ischemic damage, in contrast with all other posterior studies. Two years later, Dogan et al. [82] reported in a MCAO model that pre- and postinfusions of AM decreased the volume of ischemic brain injury, partly by increasing regional cerebral blood flow in a dose-dependent manner in rats. More recent studies have definitely proven the protective role of AM in ischemic brain. In transgenic mice that overproduce AM, the infarct area and gliosis after MCAO were reduced, in association with suppression of leukocyte infiltration, oxidative stress and apoptosis in the ischemic core. In addition, vascular regeneration and subsequent neurogenesis were enhanced, preceded by increase in mobilization of CD34+ mononuclear cells (which are endothelial cell precursors). Brain edema was also significantly reduced via suppression of vascular permeability [83]. On the other hand, Hurtado et al. [57] studied the effect of AM and its binding protein, complement factor H, in the physiopathology of stroke using a brain-specific conditional KO for AM and a complete KO for factor H. They found that animals lacking AM had got a significant infarct size increase compared with their WT littermates following middle cerebral artery occlusion. In contrast, lack of complement factor H did not affect infarct volume. They suggest that the neuroprotective action of AM in the brain may be mediated by regulation of iNOS, matrix metalloproteases, and inflammatory mediators [57]. 

Fos protein and NO-producing neurons of the rat brain, which are involved in cardiovascular regulation, are also induced by central administration of AM. Following icv administration of AM, Fos-like immunoreactive neurons were markedly increased in several brain areas of the rat, including the forebrain, the hypothalamus, and the brainstem [84, 85]. In the same conditions, the number of double-labeled neurons for Fos and NO synthase was increased in the paraventricular and supraoptic nuclei [86].

AM is hypothesized to be a physiologically relevant regulator of fluid and electrolyte homeostasis. Centrally administered AM inhibits water drinking [87] and salt appetite [88]. In addition, inactivation of endogenous AM increases water and sodium intake, suggesting that AM tonically inhibits thirst and salt intake [89]. AM may therefore participate in the central control of fluid balance by a variety of mechanisms. This peptide has direct actions in the hypothalamus to decrease vasopressin secretion and in the pituitary gland to inhibit ACTH release [90]. AM is also able to inhibit water intake after a hypovolemic challenge or a hyperosmotic challenge [87] and could diminish angiotensin II-stimulated water intake in a dose-dependent manner [90]. Another potential target for AM seems to be the area postrema which is involved in many vegetative functions, such as cardiovascular control and eating and drinking behaviour [75].

Icv injection of AM also causes a dose-dependent reduction in feeding in rats [91]. This effect is attenuated by administration of CGRP_8-37_ showing that AM action is mediated by CGRP receptors. In the same way, Martínez et al. [92] reported that AM inhibits gastric emptying in conscious rats in a dose-dependent manner by acting through adrenal-dependent, *β*-adrenergic pathways independently of central CRF receptors activation. 

Recently, AM has been reported to exert actions at each level of the hypothalamus-pituitary-adrenal (HPA) axis [93]. These data suggest that the peptide plays a role in the organization of the neuroendocrine responses to stress. AM acts within the hypothalamus of unrestrained male rats to increase HPA axis activity. AM given icv stimulates the release of prolactin but does not alter the secretion of GH, thus demonstrating the specificity of AM within the hypothalamus [94]. The role of AM in the modulation of HPA axis activity appears to be quite complex. Icv administration of AM elevated plasma corticosterone levels, which suggests that AM acts within the hypothalamus to stimulate CRH release into the hypophyseal portal vessels, thus increasing activity of the HPA axis [94]. In addition, AM has been shown to induce Fos expression in CRH-positive hypothalamic paraventricular neurons [95] and AM directly caused depolarization of parvocellular paraventricular neurons in brain slices [96]. It can be concluded that AM produced in the brain may be an important neuromodulator of the hormonal stress response. 

These results are also supported by the experiments of Fernández et al. [56]. They built a conditional KO mouse model using the Cre/loxP approach, obtaining animals with no AM nor PAMP expression in the CNS but normal levels in other organs. These genetically modified animals appear to have normal lives and do not present any gross morphological defect. Despite the normal appearance, behavioral analysis show that mice with no AM in their brain have impaired motor coordination and are hyperactive when compared to their wild-type (WT) littermates. Interestingly, heterozygous mice behave exactly as WT animals, suggesting that even a partial presence of AM during brain development is enough to prevent damage. Another characteristic of the mice lacking AM in the brain is their excessive anxiety levels. As seen before, icv administration of AM release stress hormones like corticosterone [94]. This clearly suggests that brain AM limits the magnitude of the stress response. Furthermore, KO mice had lower survival rate under hypobaric hypoxia conditions, demonstrating once again the neuroprotective function of AM in the CNS [56].

AM mRNA and protein have been shown to be expressed in dorsal root ganglia which contains nociceptors [97]. This pattern of expression has led researchers to hypothesize that AM could play a role in nociception. Ma et al. [98] demonstrated the presence of AM-like immunoreactivity in both CGRP-containing and lectin IB4-binding nociceptors in dorsal root ganglion and axon terminals in the superficial dorsal horn of the rat spinal cord. Likewise, immunoreactivity for receptor markers such as CLR and the three RAMPs is localized in the superficial dorsal horn. These results supply anatomical evidence that AM may be a pain-related neuropeptide. In addition, this group also provides functional proof that AM works as a pain neuropeptide. Moreover, intrathecal injection of AM induces a long-lasting heat hyperalgesia in rats. AM-induced pain response is mediated by a direct activation of AM receptors located on nociceptive neurons in the dorsal horn and through the activation of the PI3K/Akt/GSK3*β* signalling pathway. They also suggested that AM potentially induces the release of other pain-stimulating substances such as substance P or glutamate.

Following this line of thought, Fernández et al. [99] explored pain sensitivity in a mouse conditional KO model for AM in neurons of the CNS, including the spinal cord and dorsal root ganglia. Elimination of the AM gene in the CNS of the mouse results in expression changes for several sensory neurotransmitters, including CGRP, substance P, and enkephalin, in the dorsal root ganglia and the spinal cord. Furthermore, lack of AM expression has behavioral consequences when pain sensitivity was tested with the tail-flick and the hotplate latency paradigms. The results of the test suggest the possibility that AM acts as a nociceptive modulator in spinal reflexes, whereas it may have an analgesic function at higher cognitive levels [99]. This study confirms the important role of AM in pain sensitivity processing, but presents a more complex function of AM than previously described. 

As seen above, AM is involved in many functions in the CNS, and it also exerts some peripheral actions through CNS mechanisms. However, there is still much work to do in the area to fully understand all the roles that AM play in the nervous system. 

## 4. Adrenomedullin and Stem Cells

### 4.1. General Aspects about Stem and Progenitor Cells

Stem cells are defined by their ability to self-renew and to differentiate into many functional cell types in response to specific signals [100]. In general, stem cells produce one or more intermediate cell types before reaching the fully differentiated state which is characteristic of adult tissues. These intermediate differentiation steps are known as progenitor cells. 

During the last years, stem cells have been found in many organs and tissues, such as bone marrow, peripheral blood, umbilical cord, brain, spinal cord, dental pulp, blood vessels, skeletal muscle, gut epithelium, epidermis, cornea, retina, liver, pancreas, adipose tissue, hair follicle, mammary gland, ovary, prostate, and testis [101]. Among the main functions of these cells, the most relevant is the replacement of tissue cells which die naturally as part of tissue's homeostasis or because of an injury or illness. Stem cells are therefore indispensable for the integrity of complex and long-living organisms. 

In mammals, several stem cell niches can be found. The niche microenvironment plays a critical role in cell maintenance and differentiation since it is composed of a specialized group of cells that regulate stem cell survival, self-renewal, and differentiation [102, 103]. Strong evidence suggests now that the niche is indispensable for stem cells regulation [104]. Regulation from the niche includes growth factor signalling, cell-to-cell contact, and cell-to-extracellular-matrix interactions for homeostatic cell turnover and increased cell production in response to stimulation [105]. 

Many molecules have been described to regulate stem cell behavior. The main signals are Shh, Wnts, bone morphogenic proteins, transforming growth factor (TGF) beta, angiopoietin-1/Tie2, fibroblast growth factors, SCF/c-kit, Jagged/Notch, insulin/insulin-like growth factors, and leukemia inhibitory factor through the JAK-Stat pathway [11, 101, 106–109]. 

### 4.2. AM as a Growth and Development Factor

From the beginning, it was thought that AM was an important tumor growth factor [110]. Nowadays it is well known that AM stimulates proliferation of fibroblasts, keratinocytes, endothelial cells, osteoblasts, and many tumor-derived cells [14]. 

In Swiss 3T3 cells, AM increases DNA synthesis in a dose-dependent manner by increasing cAMP/PKA [111]. AM also stimulates DNA and cAMP synthesis in human keratinocytes [112]. These data provide evidence for a growth-promoting effect of AM, possibly mediated through cAMP. 

However, in some cell types such as VSMC, mesangial cells, glial cells, and glial tumor cells an inhibitory effect was observed for AM on proliferation and growth [14]. 

Therefore, the role of AM as a growth factor, depends on the cell type.

In addition, AM has also been proposed as an important factor in embryogenesis and differentiation [73, 113] and as an apoptosis survival factor [114]. In conclusion, there is no doubt about the role of AM in cell and tumor growth, and this might be related with a possible role of AM in the growth and differentiation of stem cells.

### 4.3. Adrenomedullin and Endothelial Progenitor Cells (EPCs)

EPCs are precursor cells which are found in circulating blood and/or in the bone marrow. EPCs are mobilized from the bone marrow into the peripheral blood in response to tissue ischemia or traumatic injury, migrating to injured endothelium sites and differentiating into mature endothelial cells *in situ*. 

Because of the main role of AM in endothelial cell biology, the number of investigations studying the role of AM in the physiology of EPCs are increasing. 

The first discoveries were that AM increases the number of early EPCs and also suppresses their apoptosis [115, 116]. AM could exert its actions on EPCs because these cells express CLR on their surface [117]. 

Nagaya et al. [116] studied the effects of AM gene-modified EPCs on the treatment of pulmonary hypertension in rats. Intravenous administration of AM-expressing EPCs significantly decreased pulmonary vascular resistance compared with EPCs alone and the AM-expressing cells ameliorated pulmonary endothelium regeneration. In addition, rats with pulmonary hypertension transplanted with AM-expressing EPCs had a significantly higher survival rate. All these results taken together suggest that AM gene transfer into EPCs inhibits cell apoptosis and induces proliferation and migration so that AM gene transfer strengthens the therapeutic potential of EPCs. 

Given that AM inhibits vascular endothelial apoptosis and induces angiogenesis, some studies investigated whether AM enhances bone marrow cell-induced angiogenesis after transplantation in a rat model of hindlimb ischemia [115]. Bone-marrow-derived mononuclear cells (MNCs) also express CLR. *In vivo*, rats which received AM infusion plus MNC transplantation showed a significantly higher laser Doppler perfusion index than the control groups and had an important improvement in blood perfusion. Also, capillary density was highest in the AM plus MNC transplantation group. The combination of AM infusion and MNC transplantation enhances MNC-induced angiogenesis. Furthermore, AM increased the number of MNC-derived von Willebrand factor-positive cells and generated *α*-SMA-positive vascular structures. The same group studied the action of AM in cultured MNCs [115]. *In vitro*, AM inhibits serum starvation-induced MNC apoptosis. The increase in MNC survival achieved by AM depends on the PI3K/Akt pathway. AM also increases MNC adhesiveness to endothelial cells via activation of adhesion molecules such as ICAM-1 and VCAM-1. MNC adhesiveness to endothelial cells is indispensable for MNC differentiation into the endothelial lineage. In addition, AM may accelerate MNC differentiation into endothelial cells since it increases the number of MNC-derived EPCs that express VE cadherin, KDR and CD31. 

The same results were obtained after transplantation of mesenchymal stem cells (MSCs) for improving neurological deficits after stroke in rats [6]. The group of animals which received AM plus MSC transplantation had a significant improvement in their health status. They also showed a marked induction of angiogenesis, and AM infusion significantly inhibited apoptosis of transplanted MSCs. 

Taken together, these data suggest that AM enhances the therapeutic potency of MNCs and MSCs through inhibition of apoptosis, induction of angiogenesis, and adhesion and differentiation improvement [6, 115].

Direct transplantation of EPCs seems to be a useful strategy for therapeutic neovascularization in ischemic tissue [117]. EPCs have been successfully used in the treatment of renal ischemia after acute kidney injury [118], myocardial regeneration [7, 119–122], and in therapeutic angiogenesis in diabetes [123]. The coadministration of AM enhances the angiogenic effects of EPCs, improves their mobilization, and helps in the development of collateral vessels [115, 116, 119, 124, 125]. In a model of hindlimb ischemia induced by resecting the right femoral artery [126], AM overexpression significantly enhanced the recovery of blood flow and increased capillary density in the ischemic leg. The results of Abe et al. [126] suggest that AM-induced angiogenesis may be associated with mobilization of bone marrow-derived cells. Treatment with AM increased bone-marrow-derived cells in the ischemic tissue, which expressed the endothelial-cell-specific protein CD31. This treatment also increased the number of Sca1 and c-kit double positive cells in peripheral blood and bone marrow. There is a possibility that the mobilization of EPCs is modulated by NO. In eNOS-deficient mice, bone marrow transplantation itself is not enough to recover the ischemic tissue [127]. It would be possible that EPC mobilization promoted by AM may be related to AM-induced NO release via activation of the PI3K/Akt pathway [126]. 

Tumors, besides recruiting neighboring blood vessels or epithelial cells, also incorporate EPCs into the developing vasculature. This process is dependent on the mobilization of VEGFR2+ and CD133+ cells into circulation. The use of an AM antagonist *in vivo* significantly reduces tumor growth and microvessel density. In tumor endothelial cells isolated from renal cell carcinoma xenografts both proliferation and migration were blocked by treatment with an AM antagonist [128]. The antagonist also reduced the number of circulating CD133+ and VEGFR2+ cells which demonstrates the importance of AM in the mobilization of EPCs [128]. 

AM is also involved in the vascular differentiation from progenitor cells. Yurugi-Kobayashi et al. [129] studied *in vitro* the acquisition of arterial or venous identity by endothelial cells induced from VEGFR2+ progenitor cells. Whereas VEGF alone mainly induced venous endothelial cells, addition of AM, which elevates cAMP, supported substantial induction of arterial endothelial cells. Stimulation of cAMP pathway induced Notch signal activation in endothelial cells. All these data indicate that coordinated signalling of VEGF, Notch, and cAMP is required to induce arterial endothelial cells from vascular progenitors. However, more research is needed to understand how arterial differentiation is achieved in the living organism. 

In the skin, sensory nerves play a main role in the pattern of arterial differentiation [130–132]. Nerves and blood vessels are branched structures. Arteries, but not veins, are specifically aligned with peripheral sensory nerves in embryonic mouse limb skin. Several lines of evidence suggest that this association reflects a requirement for the nerve to induce arterial differentiation. In mutants lacking peripheral sensory axons and/or Schwann cells, arterial differentiation does not occur and remodelling appears abnormal. Furthermore, *in vitro*, purified peripheral sensory neurons or Schwann cells can induce arterial marker expression in cultured endothelial cells [130]. The fact that this induction can be blocked by a specific VEGF antagonist suggests that nerve-promoted arteriogenesis may be mediated by local secretion of VEGF. It has also been proven that VEGF is necessary for arterial differentiation of a primitive capillary plexus *in vivo* [131]. Nerves may promote blood vessel association and arterial differentiation shortly after their arrival in the periphery in order to ensure access to a local source of survival factors during their growth. The idea that AM may be related in this process cannot be excluded, since many perivascular nerves in the rat mesenteric artery show AM-like immunoreactivity [132]. 

Very recently, it has been confirmed that AM could augment the growth and angiogenic properties of late outgrowth EPCs as potently as VEGF [133]. Hermansen et al. [134] confirmed that cultured EPCs possess an endothelial phenotype and express the AM receptor complex CLR/RAMP2. AM stimulation induced proliferation of EPCs and increased the formation of tubular networks in the EPC/fibroblast coculture and matrigel assays. These effects seem to be mediated through the PI3K/Akt signalling pathway. 

Considering all this information, it is possible to assume that AM has a main role on growth, mobilization, and differentiation of EPCs, which leads us to propose that AM may be a relevant therapeutic target for the treatment of vascular diseases. 

### 4.4. Adrenomedullin and Mesenchymal Stem Cells (MSCs)

Mesenchymal stem cells (MSCs) are adult stem cell with capacity of self-renewal and differentiation into mesoderm- and nonmesoderm-derived tissues. They also play an important role in endogenous maintenance of stem cells niches. 

There is a strong interest in searching for potential therapeutic effects of MSCs. Mobilization of endogenous MSCs represents an alternative treatment for the regeneration of injured growth plate cartilage [135]. Different aspects of MSCs render them an appropriate cell type for clinical use to promote bone regeneration [136]. Since 1995 it has been known that under *in vitro* conditions, MSCs can differentiate into cells exhibiting features of cardiomyocytes. After this seminal work, several preclinical and clinical studies have supported the notion that MSC therapy may be used for cardiac regeneration [128, 137–139]. *In vitro* works suggest that a great variety of stimuli such as insulin or fibronectin, and direct cell-to-cell contacts induce the differentiation of MSCs into cardiomyocytes. The stem cells which acquired a cardiomyocytes-like phenotype were characterized by the expression of myosin heavy chain, beta-actin, and troponin T [137]. Studies in animal models have demonstrated the ability of transplanted or infused MSCs to engraft and differentiate into cardiomyocytes, VSMCs, and endothelial cells [137, 140] and to secrete factors such as VEGF, FGF, MCP-1, HGF, IGF-1, SDF-1, thrombopoietin, and AM, to reduce tissue injury and/or enhance tissue repair [125, 138]. These factors stimulate cell adhesion, neovascularization, and formation of inflammatory infiltrate without tissue necrosis. The molecular pathways that regulate MSC-mediated regeneration are, however, poorly understood. Recently, Alfaro et al. [139] demonstrated in a murine myocardial infarct model that downregulation of the canonical Wnt pathway, characterized by significant upregulation of specific secreted frizzled-related proteins (sFRPs), was necessary for MSC self-renewal. Using a genetically modified mouse model they found that sFRP2, an inhibitor of Wnt signalling, is a key molecule for the biogenesis of a superior regenerative phenotype in MSCs. 

Several studies have investigated the role of AM in MSC transplantation. MSCs are able to secrete large amounts of angiogenic and antiapoptotic factors, including AM [125]. It has been proven that AM enhances the therapeutic potency of MSC transplantation in experimental stroke in rats [6]. AM induces angiogenesis in the ischemic tissue and significantly inhibits the apoptosis of transplanted MSCs. PI3K/Akt is an important cell survival pathway in MSCs that seems to mediate cytoprotective effects in these cells [141]. AM is able to activate the PI3K/Akt pathway, so it is reasonable to think that AM may promote MSC survival and help to cardiac repair through this pathway. 

Adipose tissue-derived stem cells (ASCs) are also adult stem cells with capacity for self-renewal and differentiation, like MSCs. Both have similar characteristics, but ASCs are closer to the endodermal and/or early hepatic differentiation stages. As with MSCs, extensive research has been done to find therapeutic roles for ASCs. Undifferentiated ASCs have the ability to improve hepatic function in mice with acute liver injury in a short-time span [142]. After the transplantation of ASCs into mice with acute liver failure, markers of liver injury decreased. In addition, injection of ASCs into the corpus cavernosum seems to be a potential therapy for the treatment of erectile dysfunction in rats with hyperlipidemia [143] and with Streptozotocin-induced diabetes [144]. This functional restoration is associated with improvements in the histology of the cavernous body, and an increased expression of VEC markers such as VE-cadherin and endothelial nitric oxide synthase. In a KO model of AM, the effect of AM-null ASCs on erectile dysfunction was significantly diminished. Furthermore, an overexpression of AM in the same cells significantly improved erectile function in the diabetic rats [144]. These results suggest that AM secreted by ASCs plays a main role in the restoration of erectile function. ASCs have also been used for the regeneration and repair of infarcted heart but its efficiency is under debate. The major problem is the poor survival rate of implanted cells. Overcoming this problem may improve stem cell therapy [145]. As AM is a factor that improves the survival in most cells, one might think of it as a way to improve ASC-induced cardiac regeneration. However, the role of AM in proliferation or apoptosis of ASCs has not been tested yet. 

### 4.5. Adrenomedullin and Hematopoietic Stem and Progenitor Cells

Hematopoietic stem cells (HSCs) possess the unique capacity to undergo self-renewal *in vivo* throughout the life of an individual while also providing the complete repertoire of mature hematopoietic and immune cells. 

Previous attempts to identify the soluble factors that regulate HSC self-renewal have been poorly successful. Chute et al. [146] demonstrated that primary human brain endothelial cells (HUBECs) support the *ex vivo* amplification of primitive human bone marrow and cord blood cells and they analyzed the gene expression of HUBEC trying to find factors with hematopoietic activity. Functional analyses demonstrated that AM synergizes with stem cell factor and Flt-3 ligand to induce the proliferation of primitive human CD34+CD38-lin-cells and promotes the expansion of CD34+ progenitors in culture. 

Apart from bone marrow, cord blood is an important source of HSCs and an alternative for allogenic transplantation. AM, which is also expressed by cord hematopoietic stem cells, has been used in combination with endothelin-1 to magnify the cord blood hematopoietic cell middle-term expansion. This effect seems to be mediated by the CLR/RAMP system [147]. AM may be used for improving the expansion of the HSC from the cord blood, which is of great importance for tissue engineering and clinical use. 

In an elastase-induced emphysema mouse model, AM infusion significantly inhibited the increase in lung volume and static lung compliance. AM also increased the number of mononuclear cells, stem cell antigen-1-positive cells in circulating blood, and the number of bone-marrow-derived cells incorporated into the elastase-treated lung [148]. Addition of AM to cultured alveolar epithelial cells and endothelial cells also attenuates elastase-induced cell death [148]. 

### 4.6. Adrenomedullin and Adrenocortical Stem Cells

The theory that adrenocortical homeostasis is maintained by undifferentiated stem or progenitor cells can be traced back nearly a century. It is thought that these rare stem cells remain relatively undifferentiated and quiescent until needed to replenish the organ. Some experiments suggest that the adrenocortical progenitors reside in the outer periphery of the adrenal gland [149]. Early microscopic studies of the adrenal glands of different vertebrates suggest the presence of peripheral undifferentiated cells containing conspicuously large euchromatin-rich nuclei. In addition, the restricted circumferential expression of preadipocyte factor 1 (Pref-1) in the glomerulosa of the rat supports the hypothesis that the glomerulosa layer contains cells on different degrees of differentiation. Moreover, experiments using radioactive thymidine indicate that subcapsular cells of the rat adrenal replenish the neighboring steroidogenic zones, suggesting that this population provides a pool of progenitors that serve to maintain the functional capacity of the cortex [149]. 

AM gene expression changes during rat adrenal regeneration after enucleation and contralateral adrenalectomy [150]. AM mRNA expression suffers a marked rise between days 1 and 5 of the regeneration. During the early stages of regeneration, local stem cells are exposed to relative hypoxia and enucleation induces an inflammatory response. Both hypoxia, via HIF, and inflammation, through NO pathways, are known to upregulate AM gene expression [62, 151]. The increase in AM gene transcription and translation may be considered one of the early events in the enucleation-induced activation of local adrenocortical stem cells. Furthermore, the two AM receptors complex, CLR/RAMP2 and CLR/RAMP3, are upregulated in enucleated adrenals, perhaps as a response to the increased local production of AM [152]. The concerted increase in AM and its receptors expression might improve the autocrine/paracrine mechanisms by which AM enhances proliferation of zona glomerulosa stem cells during adrenal regeneration. 

## 5. Adrenomedullin and Neural Stem Cells (NSCs)

The dogmatic view of an ever-immutable neural tissue in mammals is now been replaced by the notion that cell turn over, including neurons, does occur in the mature CNS throughout the adulthood, thanks to the persistence of precursor cells that possess the functional characteristics of neural stem cells (NSCs) within restricted brain areas [10]. The generation of a particular class of neuron or glial cell from a multipotent progenitor is a complex process that can be subdivided into a series of sequential steps. First, progenitor cells acquire unique positional identities through a process of spatial patterning. Multipotent progenitors produce daughter progenitor cells that are restricted to produce only one of the primary neural cell types, neurons, oligodendrocytes, or astrocytes [10]. Committed neuronal progenitors also become specialized to produce neurons of a particular kind. Neural progenitors stop dividing, migrate towards more differentiated areas, and then initiate a programme of terminal differentiation [153]. 

In the adult mammalian brain, neurogenesis occurs in the subgranular zone (SGZ) of the dentate gyrus of the hippocampus and the subventricular zone (SVZ) of the lateral ventricles [10, 154]. The SVZ is the adult brain region with the highest neurogenic rate and it represents a remnant of the embryonic germinal neuroepithelium. From the SVZ, newly generated neurons reach their destination in the olfactory bulb (OB) after a long migration through the rostral migratory stream [10]. Neurons born in the adult SGZ migrate into the granule cell layer of the dentate gyrus and become dentate granule cells [154]. Neurogenesis declines with aging in both the SVZ and the SGZ [154]. 

**Figure 1:**
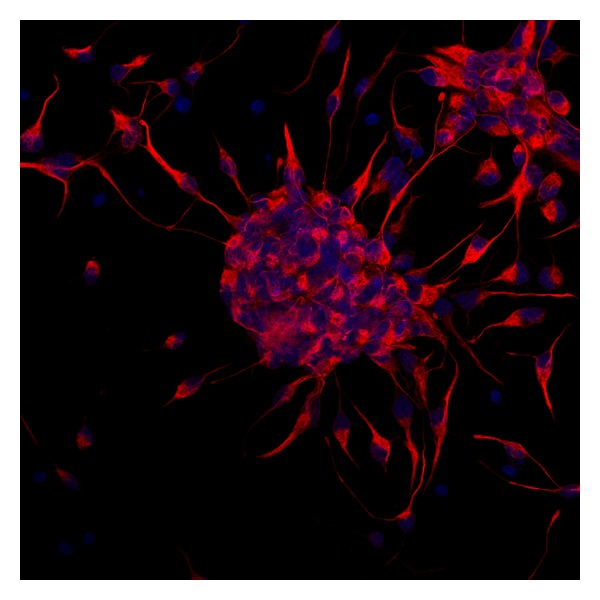
Confocal microscopy image of a neurosphere. Cells were stained with an antibody against nestin (red), a marker of stem cells, and counterstained with DAPI which labels the nuclei in blue.

Postnatal neurogenesis is subject to tight regulation, confined to isolated microenvironments, and is sensitive to neuronal activity, stress and aging. This control may be required to prevent network instability, maintain memory and behavioral patterns, and prevent tumorigenesis. Numerous researches are trying to find the molecular mechanisms that regulate NSC self-renewal, proliferation, progressive maturation, and terminal differentiation. These studies have identified many growth factors, cell signals, and transcription factors, which regulate neurogenesis [11]. After intracerebral administration of growth factors such as EGF, FGF2, or TGF-*α*, cell proliferation in the SVZ is dramatically increased and the progeny fate can change depending on the type of factor used [10]. Cultured neurospheres (Figure 1) synthesize FGF2/EGF which regulate endogenous cytokines that participate in the growth and differentiation of the neurosphere cells [155]. Some angiogenic proteins with neurotrophic effects, such as VEGF and AM, have been shown to stimulate proliferation and neuroblast production in the SVZ, while IGF-1 promotes proliferation and migration in the same niche [11]. Neurogenesis also responds to other cell signals such as neurotransmitters. While GABA and glutamate may not control the fate commitment of stem cells or progenitor cells, they could control the synthesis and release of other diffusible molecules such as growth factors. In addition, they can control the expression of transcription factors and other intracellular molecules such as microRNAs [11]. Emerging epigenetic mechanisms are critical for orchestrating nearly every aspect of neural development and homeostasis, including brain patterning, neural stem cell maintenance, neurogenesis and gliogenesis, neural subtype specification, and synaptic and neural network connectivity and plasticity [156]. Recent evidence indicates that combinations of transcription factors of the homeodomain (HD) and basic helix-loop-helix (bHLH) families establish molecular codes that determine, both where and when the different kinds of neurons and glial cells generated [157]. 

NSCs differentiate into three distinct cell types as seen above: neurons, astrocytes, and oligodendrocytes [10]. The proportion of each lineage varies considerably depending on internal and external cues. 

**Figure 2:**
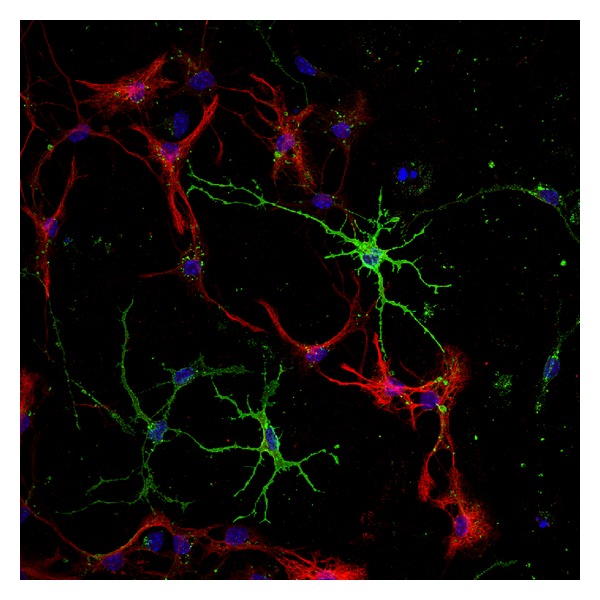
Confocal microscopy image of astrocytes and oligodendrocytes obtained from OBSC. Oligodendrocytes are stained with marker O4 (green) and astrocytes with glial fibrillary acidic protein (GFAP) (red). Cell nuclei are stained with DAPI (blue).

The generation of different cell types depends first on the presence of proneural factors, which integrate spatial and temporal cues and transform this information into neuronal subtype-specific differentiation programmes [157]. A number of progenitor proteins have been shown to select the cell types produced by progenitors of different domains in the neural tube. Among the best studied are the HD proteins Pax6 and Nk2 homeobox 2, as well as the bHLH protein oligodendrocyte transcription factor 2 (Olig2) [158]. Pax6 expression in mouse spinal cord progenitors induces the formation of neurons whereas the loss of Pax6 results in reduced neurogenesis and increased oligodendrocyte and astrocyte formation [158]. Loss of Olig2 in the mouse spinal cord results in the absence of both motor neurons and oligodendrocytes [158]. Once a progenitor cell has acquired a particular neural or glial identity, the next step is to arrest cell division and to initiate a programme of terminal differentiation [157]. Transcription factors of the bHLH family have a main role in the differentiation of neural progenitors into neurons [106]. The expression of proneural bHLH proteins, that in the mouse include Mash1, Ngn1-3, and Math1, is both necessary and sufficient to enhance the generation of differentiated neurons. Ngn2 expression in proliferating neuronal progenitors in the hippocampus support the idea that it plays a significantly role in adult neurogenesis [154]. The expression of Mash1 or Ngn2 in forebrain progenitor cells promotes the generation of GABAergic and glutamatergic neurons respectively [159]. The downstream effectors of proneural genes in the telencephalon include members of the Notch and Wnt pathways, adhesion proteins, and transduction factors [160]. Proneural genes also control later aspects of the neurogenesis process, including the arrest of progenitor division, migration of newborn neurons and terminal differentiation [157]. On the other hand, a key step in the switch of neural progenitors to gliogenesis is the induction of Sox9 (a target gene of Ngn2 [161]) and NFIA, two proteins that promote astroglial and oligodendroglial fates (Figure 2). The choice between astroglial or oligodendroglial fates is controlled by different transcription factors. The progenitor proteins Olig2 and Nkx2.2 promote oligodendrogenesis and inhibit astrogenesis [161]. The proneural proteins Mash1 is also expressed by a subset of oligodendrocyte precursors as soon as they are generated. Using transgenic mice, it has been proven that Mash1 is required for the generation of an early population of oligodendrocyte precursors in the ventral forebrain [162]. The bHLH protein SCL Tal1 has the opposite role of promoting astrogenesis by inducing astrocyte precursor-specific genes and inhibiting oligodendrogenesis via repression of Olig2 [163]. 

External cues can also modify the proportion of each lineage that is produced from the undifferentiated progenitor cells. One of these external factors is AM. The NSCs located in the OB, known as olfactory bulb stem cells (OBSCs) [164, 165], retain properties of stem cells and they can self-renew and generate neurons, astrocytes, and oligodendrocytes [166, 167]. Lack of AM results in profound changes in the proliferation rate and differentiation in the progeny of OBSCs [168]. The progeny of stem/progenitor cells isolated from the OB of AM-null mice contains a lower proportions of neurons and astrocytes and a higher proportions of oligodendrocytes than cells from WT animals. *In vitro*, this effect can be partially reversed by the addition of synthetic AM to the culture medium. These data indicate the existence of a molecular switch where AM signalling stimulates neural precursors to generate either neurons and astrocytes or oligodendrocytes [168].

Otaegi et al. [169] found that the PI3K/Akt pathway is involved in OBSC differentiation into neurons and astrocytes. As AM signalling involves the activation of Akt [170], it is possible that AM regulates the proportion of OBSC progeny through this mechanism. 

Hypoxia, via HIF-1, is known to upregulate AM gene expression [151] and, recently, it has been described that physiologic hypoxic conditions, which stimulates HIF-1 production, could strongly influence the growth of neural stem cells and their differentiation mechanisms both *in vivo* and *in vitro* [171]. Lowered oxygen tension enhances dopaminergic differentiation and survival of NSCs in a human ventral mesencephalic stem cell line. AM gene expression may be influenced by HIF-1 during NSCs growth and differentiation suggesting a possible role for AM in this process. 

Furthermore, the KO mice for AM in the CNS shows morphological changes in some areas of the brain with tubulin hyperpolymerization and an increase on Glu-tubulin immunoreactivity [56]. These data can be explained because interactions between AM and several microtubule-associated proteins (MAPs) and between PAMP and tubulin have been found [172]. Downregulation of the gene coding for both, AM and PAMP, through small interfering RNA technology results in morphological changes, microtubule stabilization, increase in posttranslational modifications of tubulin, reduction of cell motility, and partial arrest at the G2 phase of the cell cycle [172]. Cell cycle and cell migration, two cell features that require the intervention of the cytoskeleton, are very important events for all stem cells and, therefore, a defective cytoskeleton may endanger the features and functions of stem cells. Moreover, the cytoskeleton is a main point in the formation and maintenance of mature neural cells morphology and dendritic processes, and its defective function may alter both the morphology and physiology of nerve cells [173]. Although membrane receptors activated by AM and/or PAMP cannot be completely excluded, these data suggest that both peptides may have an intracellular contribution to cell growth regulation by allowing cells to pass through mitosis [172]. The AM gene-null cells show important changes in their cytoskeleton, with tubulin hyperpolymerization and changes in the actin cytoskeleton [168]. Modifications in the tubulin and actin cytoskeleton may lead to profound changes in the morphology of the stem/progenitor cells which show modifications in the cell shape and they display abundant filopodia [168]. 

Surprisingly, there are not apparent morphological changes in neurons or astrocytes derived from neurospheres lacking AM and PAMP, but the oligodendrocytes present serious modifications with shorter and less numerous cell processes. Both microtubules and microfilaments are extremely important for oligodendrocyte morphogenesis, and the differences observed in the cytoskeleton of the stem cells may be responsible for the immature phenotype described for oligodendrocytes lacking the AM gene [168]. The morphological and proliferative characteristics of the AM KO cells do not revert when synthetic AM is added to the culture medium, in contrast with what occurs with the percentage of cells of the three lineages produced by the neurospheres [168]. This suggests the existence of distinct mechanisms regulating AM-mediated stem cell differentiation and morphology. Cell fate may be mediated through the membrane receptor and the activation of the PI3K/Akt pathway, whereas morphological features and cell cycle might be more dependent on the intracellular pool of these peptides. 

**Figure 3:**
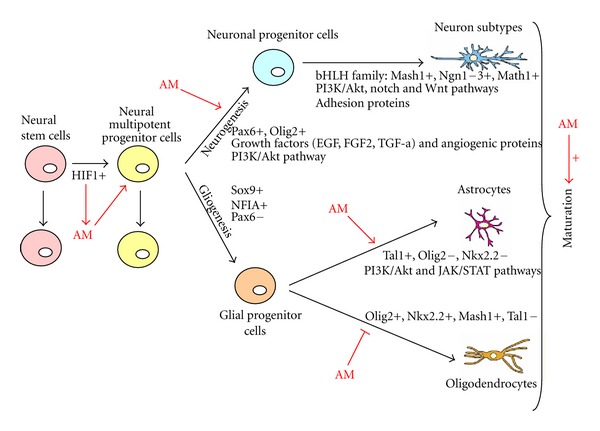
Schematic cartoon of the potential mechanisms of action of AM in NSC. AM increases the proliferation of neural multipotent progenitor cells probably through the activation of the transcription factor HIF-1. In addition, AM regulates the differentiation rate of progenitor cells into neurons, astrocytes, or oligodendrocytes. Some of these actions may be mediated by the interaction of AM and PAMP with the cytoskeleton.

Taken together, these data demonstrate that AM is an important factor in regulating the proliferation and differentiation of adult NSCs or adult neural progenitor cells (Figure 3), and that AM might be used to modulate stem cell renewal and fate in an attempt to produce and control neural stem cells for regenerative therapies.

## 6. Concluding Remarks

AM and its receptors are widely distributed throughout the CNS. AM regulates some properties of the blood-brain barrier, increases preganglionic sympathetic discharge, exerts neuroprotective actions in the brain against stroke damage, and regulates fluid balance and electrolyte homeostasis when centrally administered. Recently, it has been reported that AM can organize the neuroendocrine response to stress and play a role in nociception. 

These facts and the idea that AM acts as a growth factor and as cell fate determinant for a number of stem and progenitor cells lead us to hypothesize that AM may regulate NSC growth and differentiation. 

In this paper we have shown that AM can modify the proportion of each lineage of neurons, astrocytes, and oligodendrocytes produced from undifferentiated progenitor cells, probably through the membrane receptors for AM and the activation of the PI3K/Akt pathway. Furthermore, both AM and PAMP may be able to influence NSC proliferation, growth, and maturation through interactions with the cytoskeleton. 

AM could be used for the regulation of growth and differentiation of neural cells derived from neural progenitors as a step towards their potential therapeutic applications in the treatment of a number of neurodegenerative diseases.
